# Polyunsaturated fatty acids, inflammation, and metabolic syndrome in South Asian Americans in Maryland

**DOI:** 10.1002/fsn3.698

**Published:** 2018-07-16

**Authors:** Saira A. Khan, Robert T. Jackson

**Affiliations:** ^1^ The University of Maryland College Park MD

**Keywords:** inflammation, metabolic syndrome, n‐3 and n‐6 PUFA, South Asians

## Abstract

Metabolic syndrome (MetS) is characterized by the accumulation of cardiovascular risk factors among men and women worldwide. The use of very long‐chain polyunsaturated fatty acids (VLC PUFA) could potentially benefit individuals with MetS. The goal was to better understand the relationship between MetS and VLC PUFA in South Asian (SA) Americans who experience an elevated risk for heart disease. We analyzed a cross section of South Asian (SA) using the automated self‐administered 24‐hr recall (ASA24) and clinic data in a low‐income SA in Maryland. We found no correlation between MetS indicators (high‐density lipoprotein (HDL) cholesterol, triglycerides, fasting blood glucose, diastolic blood pressure, and waist circumference (WC)) and dietary n‐3 PUFA (eicosapentaenoic, docosapentaenoic acids). However, dietary n‐6 VLC PUFA (arachidonic acid [AA]) was associated with cholesterol and fasting blood glucose levels. SA with MetS did not have a significantly low level of dietary VLC PUFA intake, and there were no SA group differences in the intake of VLC PUFA but there were significant gender differences. Dietary practices in SA may contribute to increased proinflammatory markers and play a role in elevated MetS components.

## 1 INTRODUCTION

Metabolic syndrome (MetS) increases risk for cardiovascular disease (CVD) by twofold and type II diabetes (T2D) by fivefold through the process of insulin resistance and obesity via inflammation. However, dietary factors also play a role in MetS, especially economically disadvantaged populations who may not consume adequate amounts of healthy n‐3 polyunsaturated fatty acid (PUFA) containing foods compared to the inflammatory n‐6. The n‐3 PUFA has been associated with decreased cardiovascular death compared to n‐6, which are associated with increased inflammation. South Asian (SA) specifically tend to have high lipid abnormalities (Dodani, Henkhaus, Wick, Vacek, & Gupta, 2011; Shah, Hernandez, Mathur, Budoff, & Kanaya, 2012), dysfunction in high‐density lipoprotein (HDL) cholesterol (Dodani, Dong, Zhu, & George, 2008), and high levels of triglycerides (Chowdhury & Lasker, 2002). Aberrant lipid and glucose metabolism seem be among the key metabolic risk factors for SA groups increasing the risk for coronary heart disease leading to premature death (Cruickshank, Cooper, Burnett, MacDuff, & Drubra, 1991; Joshi et al., 2007). There is a paucity of research on these associations among the SA Americans in the United States.

Evidence suggests that modifications in dietary fat composition and ratio affect the risk of CVD, specifically, very long‐chain n‐3 (VLC n‐3) PUFA have been found to reduce plasma triglyceride levels and thereby reduce risk of CVD. Hyperlipidic diets rich in saturated fatty acids promote obesity (Roche, 2005; Warensjö, Risérus, & Vessby, 2005), insulin resistance, and MetS (Lopez‐Huertas, 2012; Melanson, Astrup, & Donahoo, 2009; Warensjö et al., 2005). A recent review summarized the effects of eicosapentaenoic (EPA) and docosahexaenoic acids (DHA) on MetS patients and showed evidence that administration of VLC n‐3 PUFA doses >1 g for at least 3 months produced a significant reduction of triglycerides ranging from 7% to 12% (Lopez‐Huertas, 2012).

Arachidonic acid (AA), an n‐6 PUFA, is the precursor of the 2‐series prostaglandins, which have greater biological activity then the 3‐series prostaglandins that are derived from the n‐3 PUFA such as EPA and DHA. A major function of AA is to serve as a precursor to the eicosanoid family of hormones that modulate immune and inflammatory responses in the body. Elevated tissue levels of AA have been associated with a number of disease states, including diabetes, obesity, and heart disease.

The objective of this study was to quantify the essential fatty acids compared to the dietary recommendations, and VLC n‐6 (lipogenic) and n‐3 (anti‐lipogenic) PUFA consumed by a cohort of understudied SA Americans by ethnicity and gender. We also examined the relationship of these PUFA and MetS components by gender. Our aim was to see whether age, gender, consumption of n‐6 and n‐3 fatty acids can predict if subjects developed MetS.

## 2 MATERIALS AND METHODS

### 2.1 Subjects

A convenience sample of 401 apparently healthy SA male and female aged between 20 and 68 years from Pakistan, India, Bangladesh, Sri Lanka, Afghanistan, Bhutan, Maldives, and Nepal defined as SA (from the CIA fact book) from two religious community medical centers in Montgomery and Baltimore Counties, Maryland were interviewed. From May 2012 to June 2013, consecutive walk‐ins were interviewed for a 24‐hr food recall in their native languages; Urdu, Panjabi, and English and the information was entered into the Automated Self‐Administered 24‐hr (ASA24) recall by the interviewer. The two centers serve as a focal point for social and religious activities and provide free medical care for low income and indigent members of the community. Subjects with metabolic disease (cancer, CVD, type 1 diabetes, HIV) were excluded.

All walk‐in patients consented to interview. Medical and chronic disease history, smoking, income categories, sociodemographic data (age, country of origin, smoking, physical activity, total years in the USA), and reason for clinic attendance were obtained. Physical activity was defined as activities above and beyond daily living (cleaning, cooking, and household chores) measured by four questions and has been described previously (Khan & Jackson, 2016).

The sample size was determined by performing a power calculation based on MetS prevalence (27%) from previous SA studies in the USA. A sample size of 360 subjects was calculated with a reasonable minimum effect of *α* = 0.05, and a minimum power 80% to detect that effect.

The subject's weight and height were measured on a Detecto Promed 6129 scale (Toledo, OH, USA) with subjects wearing light street clothing with shoes removed. Weight was measured to the nearest 0.1 kg and height to the nearest 0.1 cm. Subjects removed hair ornaments and buns from the top of the head to measure stature. Body mass index (BMI) was calculated as (weight in kg)/height in meters^2^). Waist circumference (WC) was measured at the midpoint between the iliac crest and lower rib to the nearest 0.1 cm. The cutoff values for WC were ≥90 cm males, and ≥80 cm females based on the World Health Organization (Eveleth, 1996). Information on the remaining MetS indicators was obtained from the clinic charts, and the laboratory values were analyzed by Lab Corp (Burlington, NC, USA).

### 2.2 Metabolic syndrome components

MetS was defined by the joint interim statement of the International Diabetes Federation Task Force on Epidemiology and Prevention; National Heart, Lung, and Blood Institute; American Heart Association (AHA); World Heart Federation; International Atherosclerosis Society; and International Association for the Study of Obesity as three abnormal findings out of the five: (1) WC, ≥90 cm for men, ≥80 cm for women, (2) triglycerides ≥150 m/dl or drug treatment, (3) (HDL) cholesterol, <40 mg/dl in males, <50 mg/dl in females, or drug treatment (4) elevated blood pressure or the use of hypertensive medication, systolic ≥130 and/or diastolic ≥85 mm Hg, and (5) elevated fasting glucose ≥100 mg/dl or medication.

### 2.3 24‐hr recalls

Dietary data were collected using the ASA‐24 (Subar et al., 2007). We obtained dietary information from subjects by interview and entered the data by a trained nutritionist who spoke the language (Urdu, Panjabi, and English) of the subjects. Not all SA foods were available in the database, for example, foods such as chicken curry and biryani. For these foods, we placed the individual food items (type of oil, amount of onions, garlic, ginger, etc.) into the 24 recall. The National Cancer Institute provided the methodology and the ASA24 calculated the number of food groups with the appropriate amounts of foods in each group consistent with the USDA Food Patterns with codes provided by the USDA (Blanton, Moshfegh, Baer, & Kretsch, 2006). The nutrients included in the ASA24 were total fat (g), fatty acids (total saturated (g), total monounsaturated (g), total polyunsaturated fatty acids (g), and individual fatty acids (14:0 (g), 16:0 (g), 18:0 (g), 16:1 (g), 18:1 (g), 18:2 (g), 18:3 (g), 20:4 (g),20:5 n‐3 (g), 22:5 n‐3 (g), 22:6 n‐3 (g)). Dietary fats were measured according to The World Health Organization (WHO) recommends 250 mg EPA and DHA/day for the general population (Joint, F. A. O. W. H. O. E. C. o. F. A. M., World Health, O., 2010) and the AHA recommends 2–4 g EPA and DHA/day for patients with hypertriglyceridemia and CVD (Kris‐Etherton, Harris, & Appel, 2003). The DRI for adequate intake (AI) of linolenic acid is 17 g/day for ages 31–50 years, 14 g/day >50 years for males and the AI for linoleic acid is 1.6 g/day. For females, the AI for linoleic acid is 12 g/day for ages 31–50 years, and 11 g/day for >50 years. The AI for linolenic acid is 46 g/day. These were the cutoffs that we used for this paper. The healthy eating index scores range from 0 to 100 (Table 1), and an overall score of >80 is defined as good, 51–80 needs improvement, 0–50 is poor.

**Table 1:** Components of the healthy eating index

Food group	Range of scores	Perfect score of 10^a^
1. Grains	0–10	6–11 Servings
2. Vegetables	0–10	3–5 Servings
3. Fruits	0–10	2–4 Servings
4. Milk	0–10	2–3 Servings
5. Meat	0–10	2–3 Servings
Dietary guidelines		
6. Total Fat	0–10	30% or less energy from fat
7. Saturated Fat	0–10	Less than 10% energy from saturated fat
8. Cholesterol	0–10	300 mg. or less
9. Sodium	0–10	2,400 mg. or less
10. Variety	0–10	16 different food items over 3 day period

a Note.This depends on the recommended energy intake; all amounts are listed based on a per day basis with the exception of food variety. This figure was recreated from the Center for Nutrition Policy and Promotion. http://www.cnpp.usda.gov/.

### 2.4 Statistical analysis

We performed a studentized *t*‐test between means and chi‐square between categorical data. We performed a Pearson correlation between linear data and a Spearman correlation between categorical data. Forward stepwise logistic regression was performed for those with MetS and healthy subjects. The test was considered statistically significant at *p* < 0.05.

All patients gave informed consent before participation in the study and the research protocol was approved by the Institutional Review Board (IRB) of The University of Maryland (397849‐2) and also by the review boards of the two community centers. The participants provided written consent to participate in the study and the forms were approved by the IRB prior to the study.

## 3 RESULTS

The average age of men and women was 47 and 48 years, respectively. A majority of the participants (97%) did not have medical insurance and completed 10 years. of education (90%). The income of the participants was not included as this was a clinic serving uninsured patients defined by the federal guidelines for poverty (Evaluation, O. o. t. A. S. f. P. A., 2012). We organized SA into the three largest groups based on country of origin. The other groups did not have adequate sample size for measuring differences. The three largest groups of SA were from Pakistan (*n* = 223), India (*n* = 71), and Bangladesh (*n* = 67) and did not show significant differences in the dietary intake of VLC n‐3 or n‐6 FA (Table 2). There were 41% of SA males (47%) and females (53%) that met the MetS criteria.

**Table 2:** Background characteristics of south Asian ethnic groups for metabolic syndrome components and fatty acid consumption

	Country of origin					
Characteristics	Pakistan		India		Bangladesh	
	*n* = 106	*n* = 117	*n* = 34	*n* = 37	*n* = 34	*n* = 33
	Male	Female	Male	Female	Male	Female
Age (years)	49 (11)	48 (12)	49 (11)	49 (12)	46 (11)	49 (12)
WC (cm)	97.9 (13.1)	97.2 (13.2)	96.2 (10.6)	98.2 (16.0)	94.0 (8.3)	90.6 (10.0)
Trig (μg)^b^	180.9 (131.2)	142.4 (86.3)	169.2 (116.1)	135.9 (80.1)	190.1 (135.7)	151.0 (96.4)
SBP (mmHg)	125.7 (16.4)	122.3 (19.9)	124.1 (17.2)	123.1 (20.0)	125.1 (20.9)	125.2 (22.8
DBP (mmHg)	79.0 (10.7)	75.6 (11.2)	77.4 (11.1)	74.9 (9.9)	79.2 (11.5)	77.6 (15.0)
HDL‐C (μg/ml)^a^	40.6 (10.2)	49.4 (15.3)	45.5 (16.1)	47.6 (11.6)	40.6 (9.0)	47.5 (11.1)
FBG (μg/ml)^b^	120.8 (71.3)	109.7 (38.9)	113.4 (37.2)	109.2 (47.6)	102.9 (63.2)	108.3 (37.2)
Chol (mg)	270.6 (120.2)	234.8 (179.7)	248.8 (196.4)	167.1 (158.9)	228.4 (161.3)	238.24 (180.2)
SFAT (g)	17.4 (12.4)	16.9 (9.2)	18.5 (10.9)	14.1 (7.6)	18.3 (13.2)	16.3 (10.5)
MFAT (g)	21.8 (11.5)	22.7 (12.1)	25.2 (11.2)	18.4 (10.5)	23.9 (12.1)	21.6 (11.1)
M161 (g)	0.7 (0.6)	0.7 (0.5)	0.8 (0.8)	0.5 (0.6)	0.7 (0.5)	0.7 (0.5)
M181 (g)	20.7 (10.7)	21.6 (11.7)	24.0 (10.3)	17.5 (1.0)	22.6 (11.7)	20.4 (10.9)
PFAT (g)	15.0 (10.5)	15.1 (8.0)	17.2 (8.3)	13.8 (8.6)	19.7 (9.8)	16.6 (7.4)
P182 (g)	13.1 (9.2)	13.2 (7.1)	15.1 (7.2)	11.9 (7.6)	16.8 (8.8)	13.7 (6.8)
P183 (g)	1.6 (0.1)	1.5 (0.9)	1.8 (0.9)	1.5 (1.03)	2.0 (0.9)	1.8 (0.8)
P204 (AA) (g)	0.2 (0.7)	0.2 (0.1)	0.16 (0.14)	0.1 (0.1)	0.2 (0.14)	0.2 (0.1)
P205 (EPA) (g)	0.03 (0.07)	0.04 (0.1)	0.01 (0.01)	0.04 (0.1)	0.2 (.03)	0.3 (0.5)
P225 (DPA) (g)	0.03 (0.04)	0.03 (0.05)	0.02 (0.02)	0.02 (0.03)	0.08 (0.1)	0.1 (0.2)
P226 (DHA) (g)	0.09 (0.2)	0.1 (0.2)	0.04 (0.04)	0.07 (0.2)	0.3 (0.4)	0.4 (0.8)

Notes

Chol, total cholesterol; DBP, diastolic blood pressure; FBG, fasting blood glucose; HDL‐C, high‐density lipoprotein cholesterol; MFAT, total monounsaturated fatty acids; M161, Palmitoleic Acid (n7); M181, Oleic Acid (n9); PFAT, Polyunsaturated fatty acid; P182, Linoleic Acid (n6); P183, alpha‐linolenic acid (n3); P204, Arachidonic acid (n6); P205, Eicosapentanoic acid (n6); P225, Docosapentaenoic acid (n3); P226, Docosahexaenoic acid (n3); SBP, systolic blood pressure; Trig, triglycerides; SFAT, total saturated fatty acids; WC, Waist Circumference.

a *p *<* *0.001 Significant differences between males and females.

b *p* < 0.01 between males and females. m:male/f:female.

The SA Indian males (46%) and females (62%) had the greatest differences in MetS percentage compared to other ethnic groups but no differences were found in the MetS components between the ethnic groups. The MetS components: triglycerides (*p* < 0.01), HDL cholesterol (*p* < 0.01), and fasting blood glucose (*p* < 0.001) were significantly different between males and females ((Table 2).

We compared the intake of n‐6 linoleic acid (lipogenic) and n‐3‐alpha linoleic acid (antilipogenic) to the AI requirements for SA ((Table 3). Females (69%) consumed higher percentage of n‐3‐ alpha linoleic acid compared to males (45%). AI for n‐6 linoleic acid for females was 54% compared to males (39%) ((Table 3).

**Table 3:** Adequate intake of omega‐6 linoleic acid and omega‐3 alpha linoleic Acid in South Asian male and female adults 18–70 years

	Adequate intake		Below	
	Males (*n* = 186)	Females (*n* = 204)	Males (*n* = 186)	Females (*n* = 204)
Essential fatty acids				
Omega‐6 linoleic acid	39%	54%	60%	46%
Omega‐3 alpha linoleic acid	45%	69%	55%	31%

Note

DRI for linoleic acid: males/females 31–0 years (17/12 g/day), 51–70 years (14/11 g/day); Alpha‐Linolenic Acid: males/females 31–70 years (1.6/1.1 g/day).

The MetS component fasting blood glucose (*p* < 0.05) was correlated with n‐6 AA for males and females combined. There were no correlations were found between DHA and EPA or with n‐3 alpha linoleic acid and the MetS components. Cholesterol was correlated with EPA (*p* < 0.01), DHA (*p* < 0.001) and ALA (*p* < 0.001), and fasting blood glucose (*p* < 0.05) for males but not females.

## 4 DISCUSSION

The carbon chain length and the number of double bonds are used to classify FA. FAs longer than 20 carbons are called VLC FA. FAs are classified into saturated FA (no double bonds), monosaturated FA (one double bond), and PUFA (two or more double bonds). PUFAs are further classified into n‐3 (or omega‐3 [w3]) and n‐6 (or omega 6 [w6]) series depending on the position of the terminal double bonds, that is, the double bond most distant from the carboxyl group (Abeywardena & Patten, 2011). The relative health benefits of a fatty acid depend on the sum of all its properties than whether it is short, medium, long, or very long chain. Some long‐chain FA such as n‐3 FA, alpha‐linolenic acid, DHA, and EPA‐have been shown to be very beneficial to human health. Linoleic acid (C18:2 n‐6) and alpha‐linolenic acid (C18:3 n‐3) are essential FA that must be consumed through food intake, as humans are unable to synthesize them. Less than half SA males in this study had AI of both essential FA. However, more than half of the females consumed AI of both essential FA. This raises the questions, why would there be gender differences in the dietary intake of essential FA as the meals were prepared mostly by the females at home and consumed by the entire household? Do these differences in the consumption of FA associate with MetS components either leading to or preventing inflammation complication?

We found an association between dietary AA and fasting blood glucose (*p* < 0.05) and cholesterol (*p* < 0.001) among the males in our study. FA such as the AA n‐6 are essential in small quantities but can be dangerous and pro‐inflammatory in larger amounts. The involvement of inflammatory pathway in the initiation of CVD is well‐established (Serhan & Petasis, 2011), the specific role by which inflammation contributes to its pathogenesis is not fully understood and not the topic of this study. Foods such as meat, eggs, and oils give rise to 18:2 n‐6 linoleic acid which metabolizes to AA. Prostaglandins‐2 and thromboxanes‐2 are pro‐inflammatory metabolites of AA 20:4 and associated with inflammation. Hyperglycemia and cholesterol play a role in upregulated delta desaturase. Delta desaturase is metabolized to AA which then makes the 2‐series and 4‐series mostly pro‐inflammatory mediator's lipoxgenase and cylooxygenase.

Evidence suggests that FA can affect insulin resistance, not all FA contribute equally to the process. Monounsatured fatty acid and PUFA are reported to reduce insulin resistance, while trans FA and saturated FA appear to increase insulin resistance (Benatar & Stewart, 2014). A recent Lancet study by Cruickshank et al., results suggested that the factors determining insulin secretion and is hepatic clearance in SA, could possibly include dietary fat in rates of new non‐insulin dependent diabetes mellitus. Our study also found correlations between dietary AA n‐6 and fasting blood glucose (*p* < 0.05) and triglycerides (*p* < 0.05).

Additionally, in this study, 69% of females consumed AI of the VLC n‐3 PUFA associated with therapeutic affects against cardiovascular events and only 54% males were at AI. The protective effects of estrogen combined with the consumption of n‐3 PUFA in SA females are thought to provide protective effects on the burden of disease. We did not study cardiovascular events in this study, but that may be an important factor to consider. The AHA published that non‐esterified FA was an independent risk factor for sudden death in middle‐aged males (Jouven, Charles, Desnos, & Ducimetière, 2001). An increased concentration of non‐esterified FA in the blood, absent from any known cardiovascular disease, was associated with sudden death.

Very long‐chain fatty acids is too long to be metabolized in the mitochondria so they must be metabolized by peroxisomes. However, the biosynthesis of VLC PUFAs is substantially different from the synthesis of long‐chain PUFAs, such as AA and DHA, which are synthesized in the liver from essential fatty acids obtained from dietary sources, linoleic acid (18:2 n‐6), and a‐linolenic acid (18:3 n‐3) and are subsequently transported to target tissues (Agbaga, Mandal, & Anderson, 2010).

However, it was discovered that a number of lipid mediators derived from PUFA that are endogenously generated during inflammation have potent anti‐inflammatory actions and serve as specialized pro‐solving lipid mediators and are able to promote the resolution of inflammation (Serhan, 2010; Serhan & Chiang, 2008). Additionally, while enzymatic oxygenation of AA n‐6 generates both pro‐inflammatory and pro‐solving lipid mediators, analogous pathways with the corresponding n‐3 FA EPA and DHA generally lead to the formation of anti‐inflammatory pro‐solving, cytoprotective lipid mediators (Serhan & Petasis, 2011). There was no relationship between dietary n‐3 FA consumption and the MetS components in our cohort. Some studies have shown the beneficial effects of DHA and DPA administration to patients in randomized controlled trials on LDL‐cholesterol (Ando, Sanaka, & Nihei, 1999; Egert & Stehle, 2011; Grimsgaard, Bonaa, Hansen, & Nordoy, 1997) and HDL‐cholesterol (Nestel et al., 2002), glycemic control (Woodman et al., 2002), and triglycerides (Grimsgaard et al., 1997; Park & Harris, 2003).

When evaluating the dietary intake of the SA through the 24‐hr recall, we found a majority of the group consumed red meat, chicken, yogurt, milk, and food that would give rise to the n‐6 FA as opposed to fish that are associated with the n‐3 FA. In a recent review on PUFA, it was reported that alpha linolenic acid is produce in greater quantities in seeds, nuts, and plants derivatives, and therefore should be the major n‐3 fatty acid found in humans; however, n‐6 tends to be in greater quantity in the Western diets. The main source of n‐6 fatty acids tends to be red meat, chicken, eggs, and dairy (Calder, 2013). The cross section of SA in our study consumed food items represented by the n‐6 proinflammatory pathway.

In a previous dietary analysis of SA, (Khan, Jackson, & Momen, 2016) calculated the National Cancer Institute healthy eating index scores and a fatty acid score (worth 10 points) was a part of that overall score. They reported gender differences among the fatty acid scores for those with MetS. Higher scores, meant healthier FA consumption, were found among healthy males (8.5 ± 0.6) compared to males that had MetS (7.3 ± 0.8). Females had the opposite results showing a higher FA score in MetS females (8.9 ± 0.06) compared to healthy females (7.0 ± 1.6). Evidence suggests that FA can affect insulin resistance (Mori et al., 2000; Woodman et al., 2002), not all FA contribute equally to the process. Monounsaturated fatty acid and PUFA are reported to reduce insulin resistance, while trans FA and saturated FA appear to increase insulin resistance (Mori et al., 2003). A Lancet study by Cruickshank et al. (1991), results suggested that the factors determining insulin secretion and hepatic clearance in SA, could possibly include dietary fat in rates of new non‐insulin‐dependent diabetes mellitus.

Randomized controlled trials have shown that the administration of VLC n‐3 PUFA doses of greater than one gram for at least 3 months produces a significant reduction in triglycerides ranging from 7% to 25% (Lopez‐Huertas, 2012). As we measured dietary consumption of n‐3 and n‐6 FA and did not administer purified n‐3 PFUA, we may not observe the beneficial effects of the n‐3 on triglycerides.

The role of PUFA on the burden of disease in SA leading to CVD and type II diabetes may be implicated through the diet. The n‐6 proinflammatory FA were associated with fasting blood glucose in SA. Dietary intervention and education may be valuable tools in the low‐income SA community in Maryland.

## CONFLICT OF INTEREST

The authors declare no conflict of interest or relationship, financial or otherwise.

## DATA SHARING AND DATA ACCESSIBILITY

We will make the data available to the repository.

## HUMAN SUBJECTS

This research was approved by the Internal Review Board of The University of Maryland, College Park, and the MCC Community Center Board.
